# CellExpress: a comprehensive microarray-based cancer cell line and clinical sample gene expression analysis online system

**DOI:** 10.1093/database/bax101

**Published:** 2018-01-12

**Authors:** Yi-Fang Lee, Chien-Yueh Lee, Liang-Chuan Lai, Mong-Hsun Tsai, Tzu-Pin Lu, Eric Y Chuang

**Affiliations:** 1 Graduate Institute of Biomedical Electronics and Bioinformatics, National Taiwan University, Taipei, Taiwan; 2 Graduate Institute of Physiology, National Taiwan University, Taipei, Taiwan; 3 Bioinformatics and Biostatistics Core, Center of Genomic Medicine, National Taiwan University, Taipei, Taiwan; 4 Institute of Biotechnology, National Taiwan University, Taipei, Taiwan; 5 Department of Public Health, Institute of Epidemiology and Preventive Medicine, National Taiwan University, Taipei, Taiwan

## Abstract

With the advancement of high-throughput technologies, gene expression profiles in cell lines and clinical samples are widely available in the public domain for research. However, a challenge arises when trying to perform a systematic and comprehensive analysis across independent datasets. To address this issue, we developed a web-based system, CellExpress, for analyzing the gene expression levels in more than 4000 cancer cell lines and clinical samples obtained from public datasets and user-submitted data. First, a normalization algorithm can be utilized to reduce the systematic biases across independent datasets. Next, a similarity assessment of gene expression profiles can be achieved through a dynamic dot plot, along with a distance matrix obtained from principal component analysis. Subsequently, differentially expressed genes can be visualized using hierarchical clustering. Several statistical tests and analytical algorithms are implemented in the system for dissecting gene expression changes based on the groupings defined by users. Lastly, users are able to upload their own microarray and/or next-generation sequencing data to perform a comparison of their gene expression patterns, which can help classify user data, such as stem cells, into different tissue types. In conclusion, CellExpress is a user-friendly tool that provides a comprehensive analysis of gene expression levels in both cell lines and clinical samples. The website is freely available at http://cellexpress.cgm.ntu.edu.tw/. Source code is available at https://github.com/LeeYiFang/Carkinos under the MIT License.

**Database URL**: http://cellexpress.cgm.ntu.edu.tw/

## Introduction

Cell lines play an important role as a model for conducting biological experiments and developing new therapies in cancer studies (1, 2). Theoretically, cancer cell lines show similar phenotypic features to their parental cells; however, the immortalization steps of a cell line may make the genes related to proliferation and control of the cell cycle change dramatically (3). Therefore, huge differences in gene expression profiles across different cancer cell lines exist, even if they are classified into the same organ types. Because functional assays and biological experiments usually take time and extensive effort, challenges arise when trying to select a particular cell line from one specific organ if huge discrepancies are observed. Fortunately, with the advancement of high-throughput technologies, such as microarrays and next-generation sequencing (NGS), researchers are now able to identify cancer cell lines with unusual expression patterns. To address this issue, a systematic and comprehensive system for analyzing the gene expression levels across different cell lines is required. We developed an online analytical website with a user-friendly interface, CellExpress, which simultaneously integrates >128 tissue types and 1202 cell lines from five datasets (4–7). By using the CellExpress system, researchers can easily identify cell lines that are unsuitable for further functional studies.

In addition to the intrinsic differences in cancer cell lines, the *in vitro* culturing procedure is another causative factor that affects downstream gene expression changes (8). For instance, the monolayer culturing method, which is widely used in most studies, cannot accurately mimic the growth environment of cancer cells *in vivo* (9). This is because the growth of cancer cells depends not only on the cells themselves but also the surrounding cells, including fibroblasts, pericytes, immune cells and stromal cells (10). A previous study indicated that the gene expression profile of a breast cancer cell line changed severely when compared with the profile that was obtained from its co-culturing with osteoblast cells (11). Therefore, it is difficult to directly apply the *in vivo* results identified from different individuals to *in vitro* cell line models (12). To address this issue, the CellExpress system collected the gene expression levels from both the cell lines and the clinical samples. By comparing the differences between them, researchers can identify better cell line models for advanced studies.

**Figure 1.**
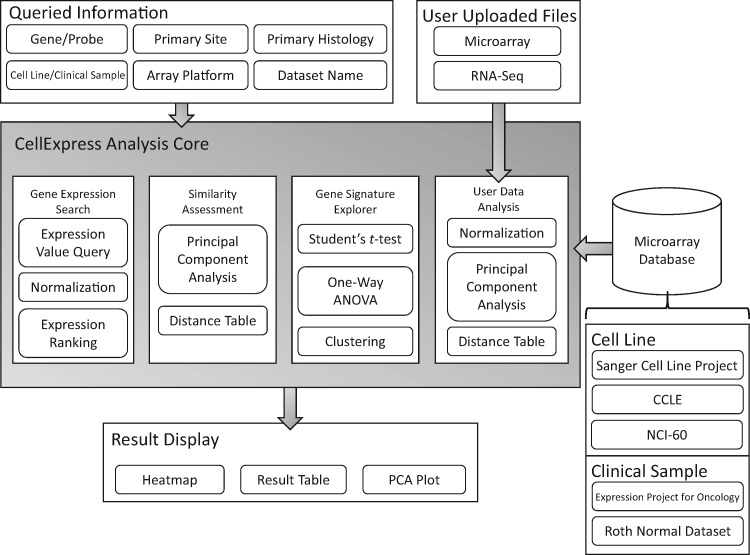
An overview of the CellExpress system. The gray block in the center shows the major functions of the CellExpress system. In the “Queried Information” block, several types of data are listed that can be searched in the CellExpress system. Both microarray and NGS data can be submitted. A total of five microarray datasets have been deposited into the CellExpress system.

Currently, several online databases are available for doing similar analysis procedures to those embedded in the CellExpress system, such as ArrayExpress (13), Gene Expression Omnibus (GEO) (14) and Cancer Cell Line Encyclopedia (CCLE) (5). However, even if all three databases contain the source files from various experiments, the barrier for biomedical researchers to obtain statistical and graphical outputs from them is very high. A user-friendly interface and an upload function are prerequisites for biomedical users to perform a comprehensive analysis. In this study, we integrated >4300 microarray slides from two widely used platforms into the CellExpress system. A comparison of deposited microarrays and an analysis of gene expression profiles provided by users can both be directly performed by the CellExpress system. In conclusion, the CellExpress system is an online system providing comprehensive analyses of gene expression levels in both cell lines and clinical samples (Figure 1).

## Materials and methods

### System implementation

The CellExpress system was written in the Python language with the Django web application framework. The front-end display was established by JavaScript and jQuery. Currently, CellExpress contains two major microarray platforms, including Affymetrix U133A and Affymetrix U133 Plus 2.0. All microarrays obtained from the same platform were normalized using the quantile normalization algorithm in order to remove systematic biases (Table 1). The control probes used for the quality check and ambiguous probes mapped to multiple genes were removed; as a result, the analysis performed in the system is based on 22,215 probes for the U133A platform and 54,613 probes for the U133 Plus 2.0 platform. We also updated and unified the names and the primary sites/histology of all cell lines in the CellExpress system based on the data from the NIH LINCS project (34) and the ExPASy (27) dataset. The reorganized cell lines are summarized in Supplementary Table S1, and the 10th revision of the International Statistical Classification of Diseases and Related Health Problems (ICD-10) coding scheme was utilized to avoid misinterpretations. In the following paragraphs, the major contents embedded in the CellExpress system are described.

**Table 1.** Datasets deposited in the CellExpress system

Dataset	Type	Number of samples	Number of cell lines	Number of tissue types	Platform	References
Sanger Cell Line Project (GSE68950)	cancer cell line	798	732	32	Affymetrix U133A	(4)
CCLE (GSE36133)	cancer cell line	917	917	24	Affymetrix U133 Plus 2.0	(5)
NCI-60 (GSE32474)	cancer cell line	174	59	9	Affymetrix U133 Plus 2.0	(6)
expO (GSE2109)	clinical sample	2152	N/A	74	Affymetrix U133 Plus 2.0	N/A
Roth Normal Dataset (GSE7307)	normal tissue	353	N/A	62	Affymetrix U133 Plus 2.0	(7)
Total (without duplication)		4394	1708 (1202)	201 (128)		

CCLE: Cancer Cell Line Encyclopedia, NCI: National Cancer Institute, expO: Expression Project for Oncology.

### Gene expression search

Overexpression and knock-down are two popular methods for characterizing the biological roles of a gene of interest. The first step to perform such experiments is to select an appropriate cell line to serve as an *in vivo* model. If an over-expression experiment is warranted, a cell line with a low endogenous expression level of the gene of interest is required. Alternatively, a cell line with a high endogenous expression level of the target gene is necessary for a knock-down experiment. A consistently expressed gene across independent cell lines is required to establish a baseline to compare the expression levels of genes of interest. Therefore, two well-known housekeeping genes, *GAPDH* and *ACTB* (28, 29), were implemented into the CellExpress system. In addition to the two housekeeping genes, we calculated the coefficient of variation (CV) for each gene in the microarray datasets, which revealed that *RPL41* had the lowest CV and a stable expression level among all datasets. Consequently, users can compare the expression level of their genes of interest in both cell lines and clinical samples after they are normalized by one of the three reference genes. Both cell lines and clinical samples in the CellExpress system can be stratified by their data sources and primary sites, and the clinical samples can be further classified based on their primary histology. The basic descriptions of the cell lines, clinical samples and normalized gene expression levels are summarized in a table and displayed in the website result page (Supplementary Figure S1), which can be retrieved directly from the CellExpress system.

### Statistical analysis of gene signature in the CellExpress system

It is important to identify differentially expressed genes based on the grouping provided by users. Therefore, two statistical tests, including the Student’s *t*-test and analysis of variance (ANOVA), were implemented in the CellExpress system to identify genes showing significant differences in the groups defined by users. The visualization of genes that are identified as differentially expressed is provided by the hierarchical clustering algorithm in the Seaborn Python package (30). Similar to the previous function, all results are summarized in a table for users to download.

### Similarity assessment of gene expression and user data analysis

To assess the similarity of gene expression levels in different microarrays, principal component analysis (PCA) is performed in the CellExpress system. The Euclidean distance was adopted to measure the quantitative differences of the genes, and all calculations were completed using the Scikit-learn Python package (31). Notably, a systematic bias may exist in the different microarrays deposited in the CellExpress system if more than two datasets were analyzed simultaneously. To address this issue, we performed a Wilcoxon rank sum test to evaluate whether the first principal component (PC1) was significantly associated with the data sources (*P* < 0.05). If a significant association was observed, the PC1 was ignored in the PCA plot. Furthermore, because the gene expression levels of a single cell line may be examined in different microarray slides from one or multiple datasets, the CellExpress system can summarize all data from the same cell line in a single value using their centroid. Lastly, the CellExpress system allows users to upload their own data to evaluate their similarity to microarrays deposited in the database. Gene expression data from both microarrays (probe-level) and NGS (gene-level) can be analyzed in the system. Either the quantile normalization algorithm or the rank invariant algorithm can be utilized to reduce systematic bias, which depends on the data type that the user submitted. Specifically, if users upload their gene expression data from the Affymetrix U133A and/or the Affymetrix U133 Plus 2.0 platforms, quantile normalization (32) will be performed. The reference baselines for quantile normalization were generated from the microarrays from the two platforms deposited in the CellExpress system. Therefore, the newly uploaded data can be compared with the gene expression data in the CellExpress system directly. However, the quantile normalization algorithm cannot be used with other microarray and NGS platforms due to the discrepancies in the number of probes and designs in the experimental systems. Therefore, the CellExpress system provides the global rank-invariant set normalization (GRSN) (33) algorithm to do the normalization in this situation. The GRSN algorithm performs normalization on the gene level instead of the probe level. In brief, for one gene targeted by multiple probes, its expression level is represented by their average value. Next, we calculate the CV for each gene from the microarrays in the CellExpress system and rank all examined genes accordingly. Therefore, the expression values serve as the GRSN normalization baseline. For real applications, we will match the gene names in the normalization baseline with those obtained from the data uploaded by users. Only the genes that are simultaneously examined in the two datasets will be retained for the advanced analyses. Subsequently, a loess regression is performed to do the normalization. Lastly, the CellExpress system provides a three-dimensional PCA plot and a summarized table for users.

## Results

### Web interface

**Figure 2.**
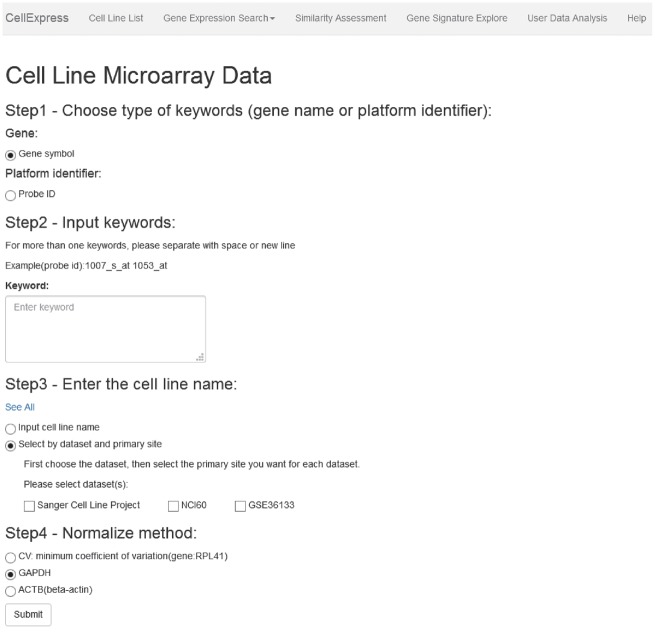
Graphical user interface of the CellExpress system. All functions are listed in the top panel. Users can follow the steps in the instructions to complete the analysis procedures.

CellExpress is a Python-based website and is now freely available at http://cellexpress.cgm.ntu.edu.tw/. As shown in Figure 2, all functions are listed in the top panel. Users can follow the steps indicated on the pages to complete the keyword input procedures. The results will be displayed in tables or figures, and all the data can be downloaded by the users.

### Overview of the major functions

The CellExpress system provides four major analytical functions: determination of expression levels, assessment of the similarity of gene expression profiles, analysis of gene signatures and comparison of user-provided data with samples in the database. Researchers can obtain the expression levels of their genes of interest across different datasets by using the gene names and/or probe names. An interactive PCA plot is embedded to help users to evaluate the similarity of the gene expression profiles in cell lines versus clinical samples. Users can explore dysregulated gene signatures in clinical samples and cell lines based on their groups of interest, such as the difference of the genetic characteristics (*TP53* wild-type versus *TP53* mutation). Both microarray and NGS data uploaded by the user can be analyzed in the CellExpress system and compared with cell lines or clinical samples deposited in the database. To demonstrate the potential applications of the CellExpress system, we have provided four examples, which are described in the following paragraphs. To show the pros and cons of both CellExpress and other online analytical systems, we have summarized the characteristics and the major functions in Table 2.

**Table 2.** Comparison of available tools for analysis of gene expression data

	Example 1	Example 2	Example 3	Example 4
CellExpress	Yes (gene expression search)	Yes (similarity assessment)	Yes (gene signature explore)	Yes (user data analysis)
CCLE	Yes (IGV)	No	Yes (differential expression analysis)	No
GEO	Limited^a^	No	Yes (GEO2R)	No
Oncomine	No^b^	No	Yes	No
ArrayExpress	No	No	No	No

CCLE: Cancer Cell Line Encyclopedia; GEO: Gene Expression Omnibus.

The modules used to analyze the examples are shown in parentheses.

a Providing a full table with probe IDs and values in each sample.

b Not supported in the free edition.

### Example 1—comparison of *ESR1* expression between ER+ and ER− cell lines

**Figure 3.**
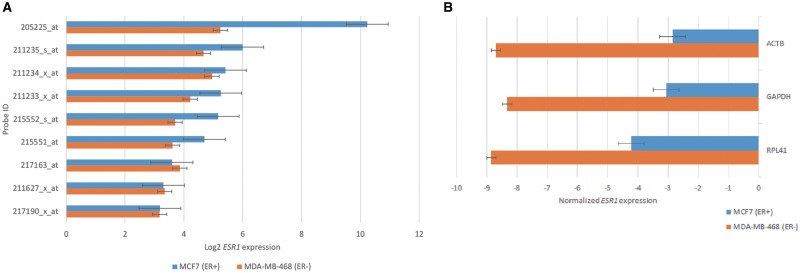
The gene expression of ESR1 in ER+ and ER− cell lines. (**A**) The ESR1 expression from nine probes in MCF7 and MDA-MB-468 cells. (**B**) The values of ESR1 expression using biological and statistical normalization methods.

A recent study reported that *ESR1* expression was significantly higher in estrogen receptor positive (ER+) patients than in estrogen receptor negative (ER−) patients (15). To evaluate whether a similar pattern can be observed in cancer cell lines, we selected MCF7 (ER+) and MDA-MB-468 (ER−) cell lines from the CCLE dataset to compare their *ESR1* expression levels. The result showed that five probes (205225_at, 211235_s_at, 211233_x_at, 215552_s_at and 215551_at) corresponding to *ESR1* had obviously higher expression values in MCF7 than in MDA-MB-468 (Figure 3A). Among the five probes, we selected the probe showing the highest gene expression level (205225_at) for further comparisons. Two housekeeping genes (*GAPDH* and *ACTB*) and one gene (*RPL41*) showing with the lowest CV across all cell lines deposited in the CellExpress system were selected as the normalization baselines. As shown in Figure 3B, a similar pattern was observed for all three normalization baselines, suggesting the efficacy of using them as the reference to compare gene expression levels from multiple datasets after normalization.

### Example 2—similarity assessment of gene expression profiles in different cell lines

**Figure 4.**
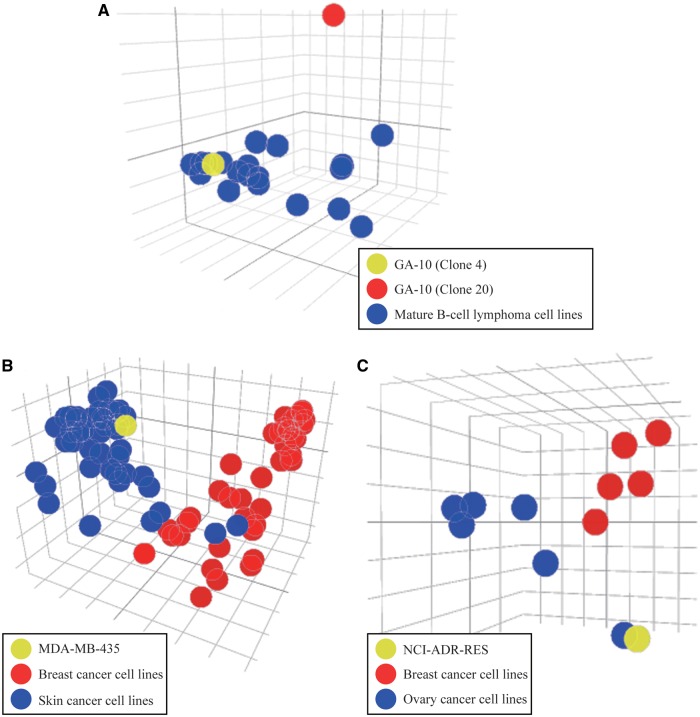
PCA plots for similarity assessment. (**A**) The PCA plot of the gene expression levels in two different clones from the GA-10 cell line and other lymphoma cancer cell lines. (**B**) The MDA-MB-435 cell line is shown in yellow and its similarity to breast (red) and skin (blue) cancer cell lines from the SCLP dataset is easily compared. (**C**) The NCI-ADR-RES cell line is shown in yellow and its similarity to breast (red) versus ovary (blue) cancer cell lines from the NCI-60 dataset is easily compared.

In this example, we used two cases to demonstrate the application of similarity assessment in the CellExpress system. First, we compared the gene expression profiles in two different clones from the GA-10 cell line (clone 4 and clone 20) with expression profiles in the other 19 mature B-cell lymphoma cell lines from the Sanger Cell Line Project (SCLP) dataset (4), since the GA-10 cell line was derived from lymphoma cells in 2001 (16). Notably, the genome-wide gene expression profile from GA-10 clone 20 showed substantial differences from GA-10 clone 4 and other lymphoma cell lines (Figure 4A). This result suggests that researchers need to use caution when using the GA-10 clone 20 as a conventional lymphoma model.

In addition to this case, two famous examples of misclassified cell lines were analyzed to examine their gene expression profiles. Initially, the breast cancer and skin cancer cell lines from the SCLP dataset were selected and their gene expression profiles were illustrated in a PCA plot (Figure 4B). Intriguingly, the MDA-MB-435 cell line, which was originally classified as a breast cancer cell line, was closer to the group of skin cancer cell lines. In support of this finding, two previous studies have indicated that the MDA-MB-435 cell line was derived from the M14 melanoma skin cell line (3, 17). Next, we analyzed the cell lines in the breast and ovary tissues from the NCI-60 dataset (6). Similar to the MDA-MB-435 cell line, the NCI-ADR-RES cell line was grouped into the cluster of ovary cancer cell lines instead of the cluster of breast cancer cell lines (Figure 4C). Three previous studies have reported that the NCI-ADR-RES cell line was isolated from the OVCAR-8 cell line (18–20), suggesting it is incorrect to classify the NCI-ADR-RES cell line as breast tissue (20). Although these two misclassification examples have been clarified in recent studies, the CellExpress system provides an efficient and simple approach to identify cell lines that are not appropriate for further use as models of a specific disease.

### Example 3—the gene signature associated with estrogen receptor expression in breast cancer

**Figure 5.**
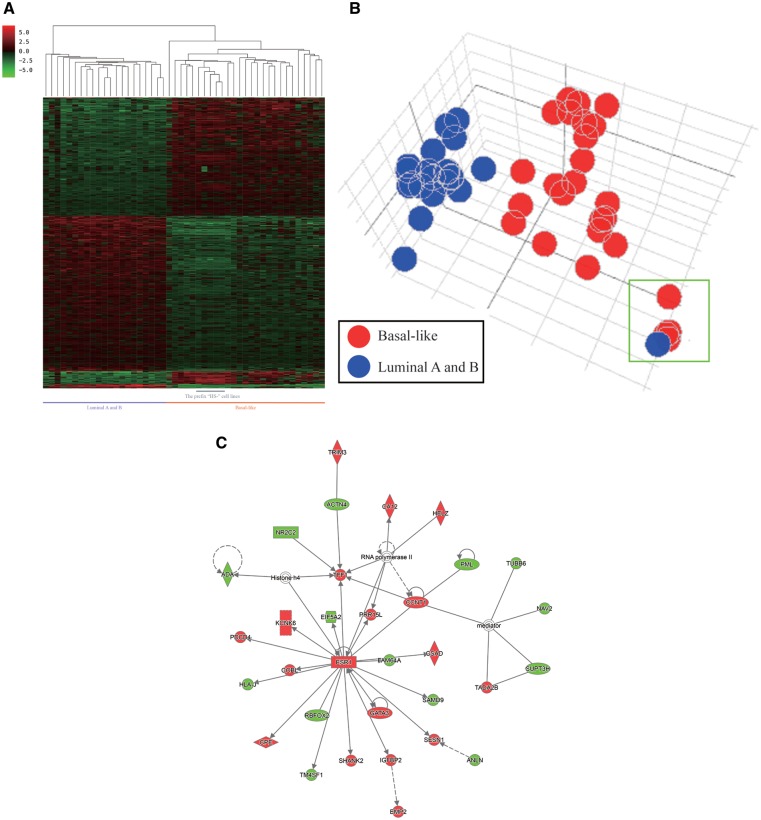
Differentially expressed genes in the basal and luminal subtypes of breast cancer cell lines from the CCLE dataset. (**A**) The heatmap of differentially expressed genes in the two subtypes. (**B**) The PCA plot of the two subtypes. The five HS cell lines (HS274T, HS281T, HS343T, HS578T and HS742T) are shown in the green rectangle. (**C**) The gene-gene interaction network of the differentially expressed genes as analyzed by IPA.

Cancer cell lines serve as an important model for identifying differentially expressed genes between the groupings specified by users, and such an approach can be performed in the CellExpress system using a few steps. For example, we selected 56 breast cancer lines from the CCLE dataset and utilized the prediction analysis of microarray 50 (PAM50) to classify the cell lines into different subtypes (21) (Supplementary Table S2). To explore the genes regulated by the estrogen receptor (ER), 21 luminal A and luminal B cell lines (ER positive) and 26 basal-like cell lines (ER negative) were further analyzed using the CellExpress system. These 47 cell lines were classified into 2 groups depending on the ER status, and a Student’s *t*-test was performed to identify differentially expressed genes. The expression levels of the top 600 significant genes were illustrated using hierarchical clustering (Figure 5A). Unsurprisingly, obvious differences between the gene expression levels can be observed in the two groups, and a similar pattern was displayed in the PCA plot (Figure 5B). Notably, the PCA plot showed that only the HS742T cell line, predicted to be the luminal A type by PAM50, was closer to the basal-like group. Intriguingly, it is worth noting that all five cell lines with an initial “HS” were clustered together in the PCA plot, suggesting their similarity in gene expression levels. Ingenuity Pathways Analysis (IPA) was performed to elucidate the affected signaling networks of the 600 differentially expressed genes. As shown in Figure 5C, the top network with the highest score was centered on the mRNA transcript of the ER (*ESR1*), which concurred with our classification using the ER status as well as several previous reports of ER positive and negative cell lines (22–24).

### Example 4—the classification of stem cells

**Figure 6.**
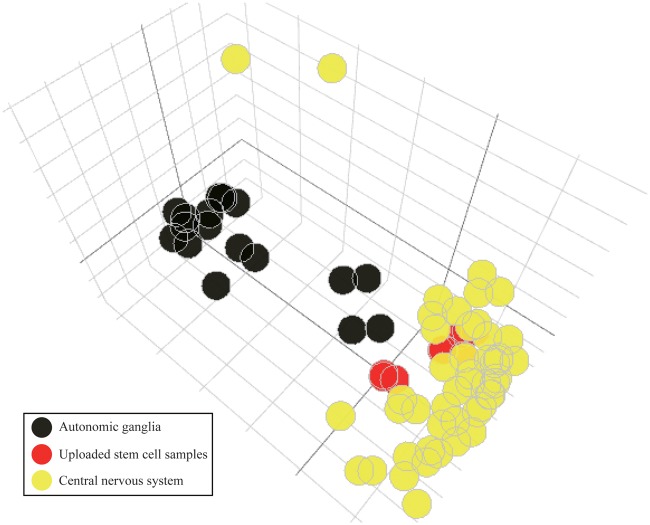
The PCA plot of stem cells and other cell lines in brain. The samples of human fetal brain neural stem cells from GSE93385 are shown in red. The cell lines from the central nervous system (yellow) and autonomic ganglia (black) are illustrated using different colors.

Lastly, we demonstrated a possible application of the CellExpress system using a stem cell study. Due to the pluripotent feature of stem cells, it is important to evaluate whether their gene expression profiles are similar to those in the targeted tissue. A human fetal brain neural stem cell dataset (GSE93385) (35) containing nine untreated RNA-Seq samples was retrieved from GEO. These nine stem cell samples were uploaded into the CellExpress system and compared with all samples in the CCLE dataset. Due to the heterogeneous sample types in the CCLE dataset, it is difficult to directly identify the tissue types showing the highest similarity to the nine stem cell samples (Supplementary Figure S2). To reduce the complexity, we selected the five cell lines from the CCLE dataset showing the nearest PCA distance to each of the stem cell samples for further comparisons (Supplementary Table S3). Notably, all selected cell lines were from the central nervous system or autonomic ganglia cells. Therefore, the cell lines of these two primary types were selected and compared with the nine stem cell samples from GSE93385. As shown in Figure 6, these stem cell samples were much closer to cell lines from the central nervous system than to cell lines from autonomic ganglia, in terms of gene expression. The results suggest that although only microarray data have been deposited in the CellExpress system, users can still analyze NGS data after performing a proper normalization algorithm. The CellExpress system is able to predict the potential tissue type of stem cells using their gene expression profiles.

## Discussion

With the rapid accumulation of high-throughput data in biomedical research fields in recent years, a comprehensive and a user-friendly analytical system can not only facilitate identification of important genes but also provide a better understanding of cellular signaling mechanisms. Considering the heterogeneity of cell lines with the same tissue type, it is difficult to select one cell line for further investigation purely based on phenotypic features. A possible solution to address this issue is to check the similarity of gene expression profiles before conducting advanced functional assays. Notably, in the misclassification examples, an unexpected result in the PCA plot of gene expression levels is not sufficient to conclude that an error in classification has occurred. For example, the two skin cancer cell lines, A431 and DJM1, were similar to the breast cancer group in Figure 4B, and currently no report has indicated that a misclassification has occurred. However, we suggest that researchers pay attention to those cell lines showing dissimilar patterns in their gene expression profiles to those of their original tissue type.

In the CellExpress system, users can easily identify differentially expressed genes based on their interested groupings and can illustrate them using hierarchical clustering and a PCA plot. In the example of ER-related genes in breast cancer cell lines (Figure 5B), the HS742T cell line was predicted to be luminal A type; however, the HS742T cell line was located within the basal-like group and was surrounded by the other four HS cell lines. A previous study indicated that some of those five HS cell lines were not from the primary tumor cells but from metastatic tumor cells (25). Therefore, their gene expression profiles were not similar to the cell lines cultured from primary tumors, and such a discrepancy should thus be taken into consideration when performing functional studies.

Until now, several online databases and analytical systems have been developed and released for usage (Table 3). We selected the top four popular systems for discussion. The Oncomine (26) system is currently the largest database with curated, annotated microarray and NGS data. However, users cannot upload their data for comparisons and payment and registration are required to access the complete data sources and perform analysis. The ArrayExpress (13) system and GEO database (14) are two popular online resources for depositing the experimental results of both microarray and NGS data. However, users cannot perform an analysis across different platforms and datasets directly on the website. Lastly, the CCLE (5) database provides a huge dataset across several molecular levels, including DNA mutations, and RNA-Seq. However, no interactive web pages/visualizations are supported in the CCLE database. Therefore, we developed the CellExpress system, which has a user-friendly interface to allow researchers to upload their own gene expression data for comparisons across different platforms, including microarrays and NGS.

**Table 3.** A comparison of online databases and analytical systems

Feature	CellExpress	CCLE	GEO	Oncomine	ArrayExpress
Platform	Microarray/NGS	Microarray	Microarray/NGS/others	Microarray/ NGS/others	Microarray/NGS
Register required	No	Yes^a^	No	Yes	No
Freeware	Yes	Yes	Yes	No	Yes
Dataset number	5	7	4348	715	69,936
Sample number	4394	1074	2,116,982	86,133	2,193,084
Cell line number	1202	917	Not provided	196	Not provided
User upload analysis	NGS data/array data	No	No	No	No
Web display	Table/cluster heatmap/ 3D PCA	Table	Table/cluster heatmap/ bar plot/distribution	Table/cluster heatmap/bar chart/box plot	Table
Figure type	Static/interactive	Static	Static	Static	N/A
Statistical analysis	Differential expression	Differential expression/ co-expression	Comparing analysis	Differential expression/ co-expression/outlier/ comparing analysis	N/A

CCLE: Cancer Cell Line Encyclopedia; GEO: Gene Expression Omnibus; NGS: next-generation sequencing.

a Registration is needed to perform customized analyses.

## Supplementary data

Supplementary data are available at *Database* online.

## Funding

This work was supported in part by the Center of Genomic Medicine, National Taiwan University, Taiwan [grant number 104R8400]; the Center of Biotechnology, National Taiwan University, Taiwan [grant number GTZ300] and the National Health Research Institutes, Taiwan [grant number NHRI-EX106-10419BI]. The funders had no role in the design, collection, analysis, or interpretation of data; in writing the manuscript; or in the decision to submit the manuscript for publication.

*Conflict of interest*. None declared. 

## Supplementary Material

Supplementary Data
