# Supplementary material for: CellExpress: a comprehensive microarray-based cancer cell line and clinical sample gene expression analysis online system
Source: Database (Oxford). 2018 Jan 12;2018:bax101. doi: 10.1093/database/bax101 (PMC7206642; doi:10.1093/database/bax101)
Supplement: Supplementary Data [file bax101_supp.doc]

**Supplementary materials**


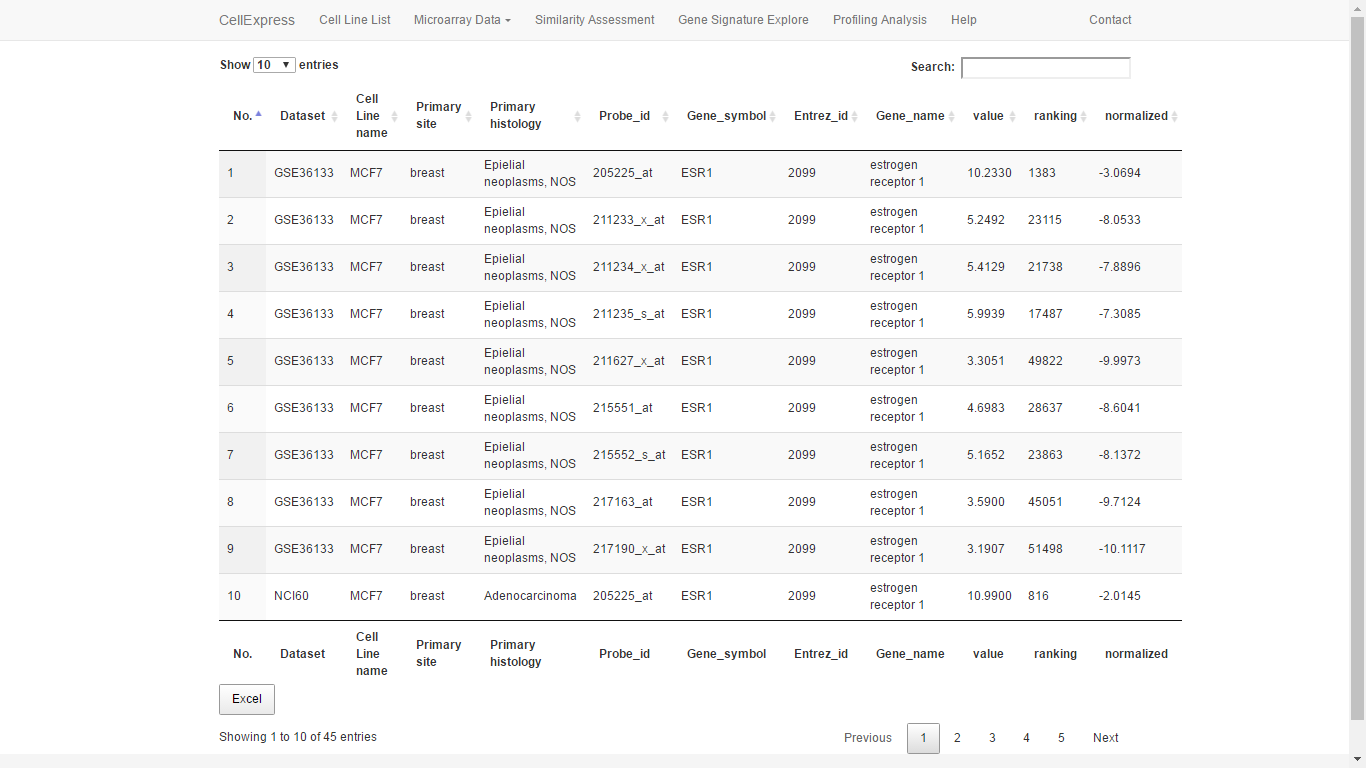


**Figure S1.** An example of search result table of *ESR1* in MCF7.


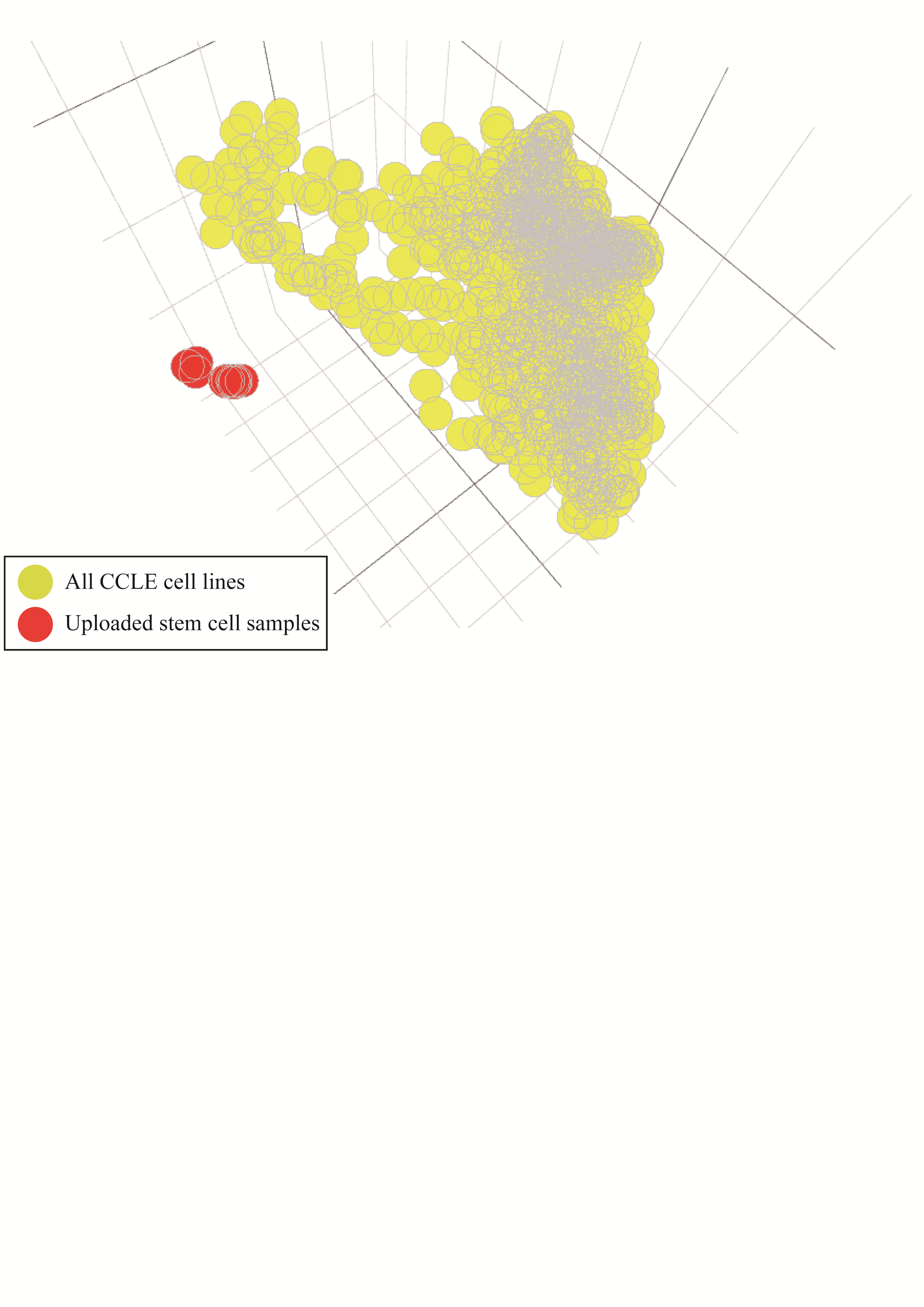


**Figure S2.** The PCA plot of all cell lines from CCLE dataset and 9 uploaded RNA-Seq samples of human fetal brain neural stem cells.

| **Table S1.** List of re-organized cell lines in the CellExpress system. | | | | |
| --- | --- | --- | --- | --- |
| Cell line name | Previous primary site | Previous primary histology | Updated primary site | Updated primary histology (ICD-10) |
| MCF7 | breast | Epithelial neoplasms, NOS | breast | Ductal, lobular and medullary neoplasms |
|  | breast | Adenocarcinoma |  |  |
| NCI-H2347 | lung | Adenocarcinoma | lung | Adenocarcinoma |
|  | lung | Epithelial neoplasms, NOS |  |  |
| KP-4 | pancreas | Adenocarcinoma | pancreas | Epithelial neoplasms, NOS |
|  | pancreas | Ductal, lobular and medullary neoplasms |  |  |
| BT-20 | breast | Adenocarcinoma | breast | Ductal, lobular and medullary neoplasms |
|  | breast | Epithelial neoplasms, NOS |  |  |
| ZR-75-30 | breast | Adenocarcinoma | breast | Ductal, lobular and medullary neoplasms |
|  | breast | Ductal, lobular and medullary neoplasms |  |  |
| A673 | bone | Miscellaneous bone tumors | bone | Miscellaneous bone tumors |
|  | soft tissue | Myomatous neoplasms |  |  |
| HPAF-II | pancreas | Adenocarcinoma | pancreas | Ductal, lobular and medullary neoplasms |
|  | pancreas | Ductal, lobular and medullary neoplasms |  |  |
| RKO | large intestine | Adenocarcinoma | large intestine | Epithelial neoplasms, NOS |
|  | large intestine | Epithelial neoplasms, NOS |  |  |
| ABC-1 | lung | Epithelial neoplasms, NOS | lung | Adenocarcinoma |
|  | lung | Adenocarcinoma |  |  |
| A549 | lung | Epithelial neoplasms, NOS | lung | Adenocarcinoma |
|  | lung | Adenocarcinoma |  |  |
| CAL-148 | breast | Adenocarcinoma | breast | Epithelial neoplasms, NOS |
|  | breast | Ductal, lobular and medullary neoplasms |  |  |
| NCI-H1838 | lung | Epithelial neoplasms, NOS | lung | Adenocarcinoma |
|  | lung | Adenocarcinoma |  |  |
| COLO-678 | large intestine | Adenocarcinoma | large intestine | Epithelial neoplasms, NOS |
|  | large intestine | Epithelial neoplasms, NOS |  |  |
| DOHH-2 | haematopoietic and lymphoid tissue | Mature B-cell lymphomas | haematopoietic and lymphoid tissue | Mature B-cell lymphomas |
|  | haematopoietic and lymphoid tissue | Malignant lymphoma |  |  |
| OVCAR-5 | ovary | Adenocarcinoma | ovary | Cystic, mucinous and serous neoplasms |
|  | ovary | Epithelial neoplasms, NOS |  |  |
| DU-4475 | breast | Epithelial neoplasms, NOS | breast | Epithelial neoplasms, NOS |
|  | breast | Ductal, lobular and medullary neoplasms |  |  |
| BT-549 | breast | Adenocarcinoma | breast | Ductal, lobular and medullary neoplasms |
|  | breast | Ductal, lobular and medullary neoplasms |  |  |
| KATOIII | stomach | Adenocarcinoma | stomach | Cystic, mucinous and serous neoplasms |
|  | stomach | Epithelial neoplasms, NOS |  |  |
| SW1573 | lung | Squamous cell neoplasms | lung | Adenocarcinoma |
|  | lung | Adenocarcinoma |  |  |
| HCC38 | breast | Adenocarcinoma | breast | Ductal, lobular and medullary neoplasms |
|  | breast | Ductal, lobular and medullary neoplasms |  |  |
| HCC1937 | breast | Adenocarcinoma | breast | Ductal, lobular and medullary neoplasms |
|  | breast | Ductal, lobular and medullary neoplasms |  |  |
| A3-KAW | haematopoietic and lymphoid tissue | Mature B-cell lymphomas | haematopoietic and lymphoid tissue | Mature B-cell lymphomas |
|  | stomach | Epithelial neoplasms, NOS |  |  |
| PSN1 | pancreas | Adenocarcinoma | pancreas | Adenocarcinoma |
|  | pancreas | Epithelial neoplasms, NOS |  |  |
| SW620 | large intestine | Adenocarcinoma | large intestine | Adenocarcinoma |
|  | large intestine | Epithelial neoplasms, NOS |  |  |
| Calu-1 | lung | Squamous cell neoplasms | lung | Squamous cell neoplasms |
|  | lung | Mucoepidermoid neoplasms |  |  |
| HOP-92 | lung | Large cell | lung | Epithelial neoplasms, NOS |
|  | lung | Epithelial neoplasms, NOS |  |  |
| CAPAN-1 | pancreas | Adenocarcinoma | pancreas | Ductal, lobular and medullary neoplasms |
|  | pancreas | Ductal, lobular and medullary neoplasms |  |  |
| SJSA-1 | bone | Osseous and chondromatous neoplasms | bone | Osseous and chondromatous neoplasms |
|  | bone | Soft tissue tumors and sarcomas, NOS |  |  |
| 22RV1 | prostate | Epithelial neoplasms, NOS | prostate | Epithelial neoplasms, NOS |
|  | prostate | Adenocarcinoma |  |  |
| SKM-1 | haematopoietic and lymphoid tissue | Myeloid leukemias | haematopoietic and lymphoid tissue | Myeloid leukemias |
|  | haematopoietic and lymphoid tissue | Myelodysplastic syndrome |  |  |
| EOL-1 | haematopoietic and lymphoid tissue | Myeloid leukemias | haematopoietic and lymphoid tissue | Chronic myeloproliferative disorders |
|  | haematopoietic and lymphoid tissue | Chronic myeloproliferative disorders |  |  |
| T84 | large intestine | Adenocarcinoma | large intestine | Epithelial neoplasms, NOS |
|  | large intestine | Epithelial neoplasms, NOS |  |  |
| TT | thyroid | Adenocarcinoma | thyroid | Epithelial neoplasms, NOS |
|  | thyroid | Ductal, lobular and medullary neoplasms |  |  |
| NCI-H1975 | lung | Epithelial neoplasms, NOS | lung | Adenocarcinoma |
|  | lung | Adenocarcinoma |  |  |
| A498 | kidney | Adenocarcinoma | kidney | Adenocarcinoma |
|  | kidney | Epithelial neoplasms, NOS |  |  |
| SR | haematopoietic and lymphoid tissue | Malignant lymphoma | haematopoietic and lymphoid tissue | Mature B-cell lymphomas |
|  | haematopoietic and lymphoid tissue | Lymphoid neoplasm |  |  |
| HCC1806 | breast | Adenocarcinoma | breast | Squamous cell neoplasms |
|  | breast | Ductal, lobular and medullary neoplasms |  |  |
| BxPC-3 | pancreas | Adenocarcinoma | pancreas | Ductal, lobular and medullary neoplasms |
|  | pancreas | Epithelial neoplasms, NOS |  |  |
| NCI-H522 | lung | Adenocarcinoma | lung | Adenocarcinoma |
|  | lung | Epithelial neoplasms, NOS |  |  |
| MKN1 | stomach | Complex epithelial neoplasms | stomach | Complex epithelial neoplasms |
|  | stomach | Epithelial neoplasms, NOS |  |  |
| HCC1187 | breast | Adenocarcinoma | breast | Ductal, lobular and medullary neoplasms |
|  | breast | Ductal, lobular and medullary neoplasms |  |  |
| Daoy | central nervous system | Miscellaneous bone tumors | central nervous system | Gliomas |
|  | central nervous system | desmoplastic |  |  |
| NCI-H2030 | lung | Epithelial neoplasms, NOS | lung | Adenocarcinoma |
|  | lung | Adenocarcinoma |  |  |
| HT-1197 | urinary tract | Epithelial neoplasms, NOS | urinary tract | Epithelial neoplasms, NOS |
|  | urinary tract | Transitional cell papillomas and carcinomas |  |  |
| RL | haematopoietic and lymphoid tissue | Mature B-cell lymphomas | haematopoietic and lymphoid tissue | Mature B-cell lymphomas |
|  | haematopoietic and lymphoid tissue | Malignant lymphoma |  |  |
| OAW-42 | ovary | Epithelial neoplasms, NOS | ovary | Cystic, mucinous and serous neoplasms |
|  | ovary | Cystic, mucinous and serous neoplasms |  |  |
| CFPAC-1 | pancreas | Adenocarcinoma | pancreas | Ductal, lobular and medullary neoplasms |
|  | pancreas | Ductal, lobular and medullary neoplasms |  |  |
| SW1990 | pancreas | Adenocarcinoma | pancreas | Adenocarcinoma |
|  | pancreas | Epithelial neoplasms, NOS |  |  |
| HT | haematopoietic and lymphoid tissue | Mature B-cell lymphomas | haematopoietic and lymphoid tissue | Mature B-cell lymphomas |
|  | haematopoietic and lymphoid tissue | Malignant lymphoma |  |  |
| EFM-19 | breast | Adenocarcinoma | breast | Adenocarcinoma |
|  | breast | Ductal, lobular and medullary neoplasms |  |  |
| MDA-MB-231 | breast | Epithelial neoplasms, NOS | breast | Adenocarcinoma |
|  | breast | Adenocarcinoma |  |  |
| HCC1599 | breast | Adenocarcinoma | breast | Ductal, lobular and medullary neoplasms |
|  | breast | Ductal, lobular and medullary neoplasms |  |  |
| MDA-MB-134-VI | breast | Adenocarcinoma | breast | Ductal, lobular and medullary neoplasms |
|  | breast | Ductal, lobular and medullary neoplasms |  |  |
| MIA-PaCa-2 | pancreas | Adenocarcinoma | pancreas | Ductal, lobular and medullary neoplasms |
|  | pancreas | Epithelial neoplasms, NOS |  |  |
| RS4-11 | haematopoietic and lymphoid tissue | Lymphoid leukemias | haematopoietic and lymphoid tissue | Lymphoid leukemias |
|  | haematopoietic and lymphoid tissue | Leukemias, NOS |  |  |
| HCC1419 | breast | Adenocarcinoma | breast | Ductal, lobular and medullary neoplasms |
|  | breast | Ductal, lobular and medullary neoplasms |  |  |
| Calu-6 | lung | Epithelial neoplasms, NOS | lung | Epithelial neoplasms, NOS |
|  | lung | Adenocarcinoma |  |  |
| T47D | breast | Adenocarcinoma | breast | Ductal, lobular and medullary neoplasms |
|  | breast | Ductal, lobular and medullary neoplasms |  |  |
| HGC-27 | stomach | Squamous cell neoplasms | stomach | Epithelial neoplasms, NOS |
|  | stomach | Epithelial neoplasms, NOS |  |  |
| UACC-812 | breast | Adenocarcinoma | breast | Ductal, lobular and medullary neoplasms |
|  | breast | Ductal, lobular and medullary neoplasms |  |  |
| TK-10 | kidney | Epithelial neoplasms, NOS | kidney | Adenocarcinoma |
|  | kidney | Adenocarcinoma |  |  |
| HCC2998 | large intestine | Epithelial neoplasms, NOS | large intestine | Adenocarcinoma |
|  | large intestine | Adenocarcinoma |  |  |
| HCC2157 | breast | Adenocarcinoma | breast | Ductal, lobular and medullary neoplasms |
|  | breast | Ductal, lobular and medullary neoplasms |  |  |
| COLO-741 | skin | Nevi and melanomas | skin | Nevi and melanomas |
|  | large intestine | Epithelial neoplasms, NOS |  |  |
| IGROV-1 | ovary | Adenocarcinoma | ovary | Adenocarcinoma |
|  | ovary | Cystic, mucinous and serous neoplasms |  |  |
|  | ovary | Epithelial neoplasms, NOS |  |  |
| HCC1143 | breast | Adenocarcinoma | breast | Ductal, lobular and medullary neoplasms |
|  | breast | Ductal, lobular and medullary neoplasms |  |  |
| OVCAR-4 | ovary | Epithelial neoplasms, NOS | ovary | Cystic, mucinous and serous neoplasms |
|  | ovary | Adenocarcinoma |  |  |
| PANC-08-13 | pancreas | Adenocarcinoma | pancreas | Ductal, lobular and medullary neoplasms |
|  | pancreas | Ductal, lobular and medullary neoplasms |  |  |
| J82 | urinary tract | Transitional cell papillomas and carcinomas | urinary tract | Epithelial neoplasms, NOS |
|  | urinary tract | Epithelial neoplasms, NOS |  |  |
| MDA-MB-157 | breast | Adenocarcinoma | breast | Epithelial neoplasms, NOS |
|  | breast | Ductal, lobular and medullary neoplasms |  |  |
| HCC2218 | breast | Adenocarcinoma | breast | Ductal, lobular and medullary neoplasms |
|  | breast | Ductal, lobular and medullary neoplasms |  |  |
| NCI-H23 | lung | Adenocarcinoma | lung | Adenocarcinoma |
|  | lung | Epithelial neoplasms, NOS |  |  |
| DB | haematopoietic and lymphoid tissue | Mature B-cell lymphomas | haematopoietic and lymphoid tissue | Mature B-cell lymphomas |
|  | haematopoietic and lymphoid tissue | Malignant lymphoma |  |  |
| HCC1954 | breast | Adenocarcinoma | breast | Ductal, lobular and medullary neoplasms |
|  | breast | Ductal, lobular and medullary neoplasms |  |  |
| A4-Fuk | haematopoietic and lymphoid tissue | Mature B-cell lymphomas | haematopoietic and lymphoid tissue | Lymphoid leukemia |
|  | skin | Nevi and melanomas |  |  |
| HS578T | breast | Adenocarcinoma | breast | Ductal, lobular and medullary neoplasms |
|  | breast | Complex mixed and stromal neoplasms |  |  |
|  | breast | Epithelial neoplasms, NOS |  |  |
| G-401 | soft tissue | Complex mixed and stromal neoplasms | kidney | Complex mixed and stromal neoplasms |
|  | kidney | Complex mixed and stromal neoplasms |  |  |
| DU-145 | prostate | Epithelial neoplasms, NOS | prostate | Epithelial neoplasms, NOS |
|  | prostate | Adenocarcinoma |  |  |
| NCI-N87 | stomach | Epithelial neoplasms, NOS | stomach | Adenocarcinoma |
|  | stomach | Adenocarcinoma |  |  |
| RERF-LC-MS | lung | Epithelial neoplasms, NOS | lung | Adenocarcinoma |
|  | lung | Adenocarcinoma |  |  |
| PC-14 | lung | Epithelial neoplasms, NOS | lung | Adenocarcinoma |
|  | lung | Adenocarcinoma |  |  |
| HCC1395 | breast | Adenocarcinoma | breast | Ductal, lobular and medullary neoplasms |
|  | breast | Ductal, lobular and medullary neoplasms |  |  |
| MDA-MB-435 | skin | Ductal, lobular and medullary neoplasms | skin | Nevi and melanomas |
|  | breast | Epithelial neoplasms, NOS |  |  |
| MSTO-211H | pleura | Mesothelial neoplasms | pleura | Mesothelial neoplasms |
|  | pleura | biphasic |  |  |
| PANC-03-27 | pancreas | Adenocarcinoma | pancreas | Adenocarcinoma |
|  | pancreas | Ductal, lobular and medullary neoplasms |  |  |
| MDA-MB-175-VII | breast | Adenocarcinoma | breast | Ductal, lobular and medullary neoplasms |
|  | breast | Ductal, lobular and medullary neoplasms |  |  |
| HCT-15 | large intestine | Adenocarcinoma | large intestine | Adenocarcinoma |
|  | large intestine | Epithelial neoplasms, NOS |  |  |
| AsPC-1 | pancreas | Adenocarcinoma | pancreas | Ductal, lobular and medullary neoplasms |
|  | pancreas | Epithelial neoplasms, NOS |  |  |
| NCI-ADR-RES | ovary | Adenocarcinoma | ovary | Cystic, mucinous and serous neoplasms |
|  | breast | Epithelial neoplasms, NOS |  |  |
| HT-29 | large intestine | Adenocarcinoma | large intestine | Adenocarcinoma |
|  | large intestine | Epithelial neoplasms, NOS |  |  |
| HCC70 | breast | Adenocarcinoma | breast | Ductal, lobular and medullary neoplasms |
|  | breast | Ductal, lobular and medullary neoplasms |  |  |
| NCI-H1703 | lung | Adenocarcinoma | lung | Squamous cell neoplasms |
|  | lung | Complex epithelial neoplasms |  |  |
| PANC-10-05 | pancreas | Adenocarcinoma | pancreas | Adenocarcinoma |
|  | pancreas | Ductal, lobular and medullary neoplasms |  |  |
| NCI-H1793 | lung | Epithelial neoplasms, NOS | lung | Adenocarcinoma |
|  | lung | Adenocarcinoma |  |  |
| OVCAR-3 | ovary | Epithelial neoplasms, NOS | ovary | Cystic, mucinous and serous neoplasms |
|  | ovary | Adenocarcinoma |  |  |
| ICD-10: The 10th revision of the International Statistical Classification of Diseases and Related Health Problems | | | | |

| **Table S2.** Classification results of breast cancer cell lines in the CCLE dataset by PAM50 | | |
| --- | --- | --- |
| **Sample Name** | **Cell Line** | **PAM50 Result** |
| GSM886891 | BT-20 | Basal |
| GSM886894 | BT-549 | Basal |
| GSM886904 | CAL-120 | Basal |
| GSM886909 | CAL-51 | Basal |
| GSM886913 | CAL-85-1 | Basal |
| GSM886989 | DU-4475 | Basal |
| GSM887033 | HCC1143 | Basal |
| GSM887035 | HCC1187 | Basal |
| GSM887037 | HCC1395 | Basal |
| GSM887041 | HCC1569 | Basal |
| GSM887042 | HCC1599 | Basal |
| GSM887044 | HCC1806 | Basal |
| GSM887045 | HCC1937 | Basal |
| GSM887048 | HCC2157 | Basal |
| GSM887054 | HCC38 | Basal |
| GSM887058 | HCC70 | Basal |
| GSM887066 | HDQ-P1 | Basal |
| GSM887094 | HS274T | Basal |
| GSM887095 | HS281T | Basal |
| GSM887097 | HS343T | Basal |
| GSM887098 | HS578T | Basal |
| GSM887183 | JIMT-1 | Basal |
| GSM887293 | MDA-MB-157 | Basal |
| GSM887295 | MDA-MB-231 | Basal |
| GSM887299 | MDA-MB-436 | Basal |
| GSM887301 | MDA-MB-468 | Basal |
| GSM886871 | AU565 | Her2 |
| GSM886998 | EFM-192A | Her2 |
| GSM887046 | HCC1954 | Her2 |
| GSM887047 | HCC202 | Her2 |
| GSM887300 | MDA-MB-453 | Her2 |
| GSM887575 | SK-BR-3 | Her2 |
| GSM886892 | BT-474 | LumA |
| GSM886893 | BT-483 | LumA |
| GSM886906 | CAL-148 | LumA |
| GSM886917 | CAMA-1 | LumA |
| GSM886999 | EFM-19 | LumA |
| GSM887038 | HCC1419 | LumA |
| GSM887040 | HCC1500 | LumA |
| GSM887049 | HCC2218 | LumA |
| GSM887114 | HS742T | LumA |
| GSM887238 | KPL-1 | LumA |
| GSM887291 | MCF7 | LumA |
| GSM887292 | MDA-MB-134-VI | LumA |
| GSM887294 | MDA-MB-175-VII | LumA |
| GSM887296 | MDA-MB-361 | LumA |
| GSM887297 | MDA-MB-415 | LumA |
| GSM887727 | UACC-812 | LumA |
| GSM887728 | UACC-893 | LumA |
| GSM887749 | YMB-1 | LumA |
| GSM887751 | ZR-75-30 | LumA |
| GSM887039 | HCC1428 | LumB |
| GSM887685 | T47D | LumB |
| GSM887750 | ZR-75-1 | LumB |
| GSM887101 | HS606T | Normal |
| GSM887113 | HS739T | Normal |

| **Table S3.** The 5 cell lines in the CCLE dataset showing the shortest PCA distance to each RNA-Seq sample in GSE93385. | | | | |
| --- | --- | --- | --- | --- |
| **Sample ID** | **Cell line** | **Primary site of the cell line** | **Primary histology of the cell line** | **Distance** |
| SRR5164629 | KNS-42 | central nervous system | Gliomas | 170.14 |
|  | SNU-201 | central nervous system | Gliomas | 170.92 |
|  | MHH-NB-11 | autonomic ganglia | Neuroepitheliomatous neoplasms | 171.37 |
|  | SNU-626 | central nervous system | Gliomas | 172.02 |
|  | SNU-466 | central nervous system | Gliomas | 172.72 |
| SRR5164630 | KNS-42 | central nervous system | Gliomas | 169.60 |
|  | MHH-NB-11 | autonomic ganglia | Neuroepitheliomatous neoplasms | 170.13 |
|  | SNU-201 | central nervous system | Gliomas | 170.62 |
|  | SNU-626 | central nervous system | Gliomas | 171.60 |
|  | SNU-466 | central nervous system | Gliomas | 172.56 |
| SRR5164631 | KNS-42 | central nervous system | Gliomas | 161.40 |
|  | MHH-NB-11 | autonomic ganglia | Neuroepitheliomatous neoplasms | 162.27 |
|  | SNU-201 | central nervous system | Gliomas | 162.38 |
|  | SNU-626 | central nervous system | Gliomas | 163.36 |
|  | SNU-466 | central nervous system | Gliomas | 164.31 |
| SRR5164635 | KNS-42 | central nervous system | Gliomas | 160.45 |
|  | SNU-201 | central nervous system | Gliomas | 160.54 |
|  | SNU-626 | central nervous system | Gliomas | 161.95 |
|  | SNU-466 | central nervous system | Gliomas | 161.96 |
|  | U-251-MG | central nervous system | Gliomas | 163.62 |
| SRR5164636 | SNU-201 | central nervous system | Gliomas | 162.38 |
|  | KNS-42 | central nervous system | Gliomas | 162.62 |
|  | SNU-466 | central nervous system | Gliomas | 163.71 |
|  | SNU-626 | central nervous system | Gliomas | 164.03 |
|  | U-251-MG | central nervous system | Gliomas | 165.63 |
| SRR5164637 | KNS-42 | central nervous system | Gliomas | 162.02 |
|  | SNU-201 | central nervous system | Gliomas | 162.16 |
|  | SNU-626 | central nervous system | Gliomas | 163.54 |
|  | SNU-466 | central nervous system | Gliomas | 163.60 |
|  | U-251-MG | central nervous system | Gliomas | 165.23 |
| SRR5164641 | SNU-201 | central nervous system | Gliomas | 158.24 |
|  | KNS-42 | central nervous system | Gliomas | 158.25 |
|  | SNU-466 | central nervous system | Gliomas | 159.60 |
|  | SNU-626 | central nervous system | Gliomas | 159.67 |
|  | U-251-MG | central nervous system | Gliomas | 161.31 |
| SRR5164642 | SNU-201 | central nervous system | Gliomas | 153.38 |
|  | KNS-42 | central nervous system | Gliomas | 153.53 |
|  | SNU-466 | central nervous system | Gliomas | 154.68 |
|  | SNU-626 | central nervous system | Gliomas | 154.89 |
|  | U-251-MG | central nervous system | Gliomas | 156.49 |
| SRR5164643 | KNS-42 | central nervous system | Gliomas | 155.62 |
|  | SNU-201 | central nervous system | Gliomas | 155.64 |
|  | SNU-466 | central nervous system | Gliomas | 157.02 |
|  | SNU-626 | central nervous system | Gliomas | 157.05 |
|  | U-251-MG | central nervous system | Gliomas | 158.70 |
| PCA: Principal Component Analysis; CCLE: Cancer Cell Line Encyclopedia  Only the top 5 of the shortest distances in each sample of neural stem cell are shown. | | | | |
